# Identification of feature risk pathways of smoking-induced lung cancer based on SVM

**DOI:** 10.1371/journal.pone.0233445

**Published:** 2020-06-04

**Authors:** Rongjun Chen, Jinhui Lin

**Affiliations:** 1 Department of Surgery, Anji Third People’s Hospital, Zhejiang China; 2 Department of Radiology, Taizhou Municipal Hospital, Zhejiang China; Chinese Academy of Sciences, CHINA

## Abstract

**Objective:**

The present study aims to explore the role of smoking factors in the risk of lung cancer and screen the feature risk pathways of smoking-induced lung cancer.

**Methods:**

The expression profiles of the patient data from GEO database were standardized, and differentially expressed genes (DEGs) were analyzed by limma algorithm. Samples and genes were analyzed by Unsupervised hierarchical clustering method, while GO and KEGG enrichment analyses were performed on DEGs. The data of the protein-protein interaction (PPI) network were downloaded from the BioGrid and HPRD databases, and the DEGs were mapped into the PPI network to identify the interaction relationship. The enriched significant pathways were used to calculate the anomaly score and RFE method was used to optimize the feature sets. The model was trained using the support vector machine (SVM) and the predicted results were plotted into ROC curves. The AUC value was calculated to evaluate the predictive performance of the SVM model.

**Results:**

A total of 1923 DEGs were obtained, of which 826 were down-regulated and 1097 were up-regulated. Unsupervised hierarchical clustering analysis showed that the diagnosis accuracy of lung cancer smokers was 74%, and that of non-lung cancer smokers was 75%. Five optimal feature pathway sets were obtained by screening, the clinical diagnostic ability of which was detected by SVM model with the accuracy improved to 84%. The diagnostic accuracy was 90% after combining clinical information.

**Conclusion:**

We verified that five signaling pathways combined with clinical information could be used as a feature risk pathway for identifying lung cancer smokers and non-lung cancer smokers and increased the diagnostic accuracy.

**Highlights**

1. 1923 differentially expressed genes between lung cancer smokers and non-lung cancer smokers were excavated and used for functional enrichment analysis.
2. Unsupervised hierarchical clustering method was used for cluster analysis of samples. The diagnosis accuracy of lung cancer was 74% and that of non-lung cancer was 75%.
3. A total of five dominant pathways were obtained by feature pathway screening. The diagnostic value of these feature pathways was identified based on SVM, and the diagnostic accuracy was improved to 84% which reached 90% after combining clinical information.

## Introduction

Lung cancer is the most common cancer in the respiratory system with high malignancy and extremely fast progression. According to 2018 Global cancer statistics (CA), lung cancer accounts for 11.6% of all types of cancers worldwide, and is also the leading cause of cancer deaths (accounting for 18.4% of total cancer deaths) [1]. Numerous studies have shown that lung cancer has a higher incidence in male and is significantly associated with long-term smoking [2, 3]. Lung cancer often begins in the periphery of lung tissue without significant clinical features in early stage. In the advanced stage, it is accompanied by chronic cough, blood stasis, chest tightness and other symptoms. In this case, some early symptoms such as fatigue, shortness of breath, upper back and chest pain are likely to be ignored, resulting in poor therapeutic effect.

Smoking is the leading cause of lung cancer and a global factor in many other cancer deaths. In developing countries, the proportion of smokers has gradually increased. While in developed countries, there are relatively few smokers. In the United States, patients with a history of smoking amounting for 10–15% of the total lung cancer patients [4]. Although most lung cancer patients have a long history of smoking, the mechanism of lung cancer in non-smokers remains unclear. At the same time, people who smoke for a long time but do not have lung cancer also account for a considerable proportion [5]. Therefore, the specific biological mechanism of smoking as an inducing factor leading to lung cancer needs to be verified, which would be of great significance to clinically diagnose the risk of lung cancer. Studies have shown that the mutation in related genes is one cause for smoking-induced lung cancer. Chapman AM *et*. *al* have found high frequency mutations of EGFR, EML4, ALK and KRAS in the lung cancer patients with a smoking history [4]. The widely studied tumor suppressor gene p53 is also discovered to be an important role in the process of smoking-induced lung cancer. Gibbons DL *et*. *al* have reported that tobacco-induced p53 mutation may be the main pathway of the smoking-induced lung cancer development by *in vivo* and *in vitro* experiments [6]. Lee PN *et*. *al* have discovered a strong correlation between smoking and carcinogenesis of lung cancer through analysis of 287 studies. The dose of smoking is significantly related to risks of all types of lung cancer [7].

With the continuous development of gene sequencing technology, molecular level studies are important for explaining the pathogenesis of cancer, identifying potential targets for new drugs, and screening clinical biomarkers. The second-generation sequencing technology, gene chip and other technical means can be applied to clinical cancer research. There are many studies explaining the possible pathogenesis of lung cancer and identifying lung cancer-related genes through gene expression profiles. Wei D *et*. *al* have analyzed gene expression profiles and identified a variety of lung cancer-related genes associated with lymph node metastasis, tumor TNM staging, patient survival and so an [8]. In addition, molecular-level features could help us assess patient sensitivity to treatment. For example, Hamilton G *et*. *al* have found that the expression of chitinase-3-like 1/YKL-40 is significantly associated with drug resistance in patients. Additionally, by early detection of molecular level expression, the risk of chemotherapy failure for patients can be assessed in advance, avoiding side effects and treatment delay [9].

Most studies focus on smoking as a major inducer of lung cancer and are devoted to identifying lung cancer-related genes and explaining its biological mechanisms. However, few studies have focused on non-lung cancer in long-term smokers. It is concluded that smoking is not the only cause of lung cancer. There are more complex high-dimensional interactions in the development of lung cancer. Some feedback and antagonism of human activation under tobacco stimulation may be the reason why these smokers are free from lung cancer.

Support vector machine (SVM) learning is a powerful classification tool, which has been used in cancer genome classification or subtyping. Nowadays, with the progress of high-throughput technology, a large number of genomic and epigenomic data have been generated. The classification characteristics of SVM are expanding its application in cancer genomics to find new biomarkers, new drug targets, and a better understanding of cancer driver genes [10]. Therefore, we used bioinformatics methods to compare two smoker groups with or without lung cancer to identify differences in molecular levels, and to establish a diagnostic model based on SVM.

## Materials and methods

### Data collection and preprocessing

GSE4115 dataset was downloaded from Gene Expression Omnibus (GEO) database (https://www.ncbi.nlm.nih.gov/geo/) which contained 192 samples, including 97 samples from lung cancer smokers, 90 samples from non-lung cancer smokers and 5 samples from suspicious lung cancer smokers. In addition, clinical information including age, gender, duration of smoking, smoking index, tumor size and with or without lymphadenopathy were collected for further analysis. 163 samples with completely clinical characteristics were screened out, including 85 samples of non-lung cancer smokers and 78 samples of lung cancer smokers. The expression profiles were tested through Affymetrix Human Genome U133A Array platform. All probe IDs were transferred to gene symbols according to platform data (GPL96-15653.txt). Since multiple probes might correspond to the same gene symbol, the results of the same gene symbol were averaged and merged. In order to eliminate the effect of differences in intrinsic expression level between genes, the expression value of all genes was normalized according to Z-score [11], which is to calculate the mean and standard deviation of expression in all samples for each gene symbol, and finally correct the expression value X for each sample. X’ = (X-mean)/SD (X’ is the corrected expression value).

### Extraction of Differentially Expressed Genes (DEGs)

Differential analysis was performed using “limma” [12] R package. The smoking patients with lung cancer (lung cancer smokers) were in case group, while smoking patients without lung cancer (non-lung cancer smokers) were in control group. Since the logFC of most genes concentrated between -1 and 1, a gene with *p* value less than 0.01 after FDR correction was regarded as a significantly DEG to ensure statistical efficiency.

### Hierarchical cluster analysis

Unsupervised clustering analysis, namely hierarchical cluster analysis, was performed and visualized by heat map. The clustering process was implemented by orange software and visualized by distance map. Pearson correlation coefficient [13], average linkage [14] and hierarchical clustering method [15] were adopted for cluster analysis.

### Functional enrichment analysis

The Database for Annotation, Visualization and Integrated Discovery (DAVID) [16] was used to perform Gene Ontology (GO) functional and Kyoto Encyclopedia of Genes and Genomes (KEGG) pathway enrichment analyses for up-regulated and down-regulated DEGs, respectively. Terms with significant P<0.05 after hypergeometric test were selected as significantly related terms with GO and KEGG.

### Protein-Protein Interaction (PPI) network construction

The DEGs were mapped to the human PPI network to identify the intrinsic association or the importance of disease-related genes in the system network. The data of the PPI were downloaded from the BioGrid [17] (https://thebiogrid.org) and HPRD [18] (http://www.hprd.org/) databases. The two sets of data were combined into a union, including 14,553 proteins and 662,360 interactions. Next, DEGs were mapped into a human PPI to identify interactions. Cytoscape was used to build network and conduct visualize analysis. Meanwhile, the network analysis plug-in was used to analyze the network topological features in order to identify the hub nodes with high node degree in network.

### Calculation of functional deviation score matrix

The abnormal score was calculated using the enriched significant pathway. Changes of each pathway under each sample were obtained by calculating the expression values of the DEGs in the significant pathway. The formula is as follows:

$$pathscore=log\frac{\sqrt{\sum_{i}^{m}\omega_{i}{\left(d_{i}-\bar{d_{i}}\right)}^{2}}}{\sqrt{\sum_{j}^{n}\omega_{j}{\left(d_{j}-\bar{d_{j}}\right)}^{2}}}$$

Assuming that there were m up-regulated genes and n down-regulated genes enriched in pathway P, and $\bar{d}$ was the mean of up-regulated genes i or down-regulated genes j in a non-lung cancer smoker. Euclidean distance was used to calculate the deviation of pathway P under the influence of up-regulated genes and down-regulated genes, respectively (ω is the node degree of genes in the PPI as a weight to evaluate contribution rate). The logarithm transformation value of log was taken. The pathway function was significantly up-regulated in lung cancer smokers compared to non-lung cancer smokers when the score of P pathway was greater than 0. When the score was less than 0, opposite results were observed.

### Recursive Feature Elimination (RFE) feature extraction

We integrated all pathway feature sets and clinical characteristics data, including age, gender, smoking age, smoking index, tumor size, presence or absence of lymph node lesions, etc., and obtained a set of features consisting of clinical diagnostic indicators and abnormal pathways. RFE [19] was used to perform feature set optimization screening. All features were constituted into multiple small feature sets and combined cross-validation to iteratively test the training set. At least k significant features were removed each time until the best prediction accuracy was obtained.

### SVM classifier construction based on optimized features

The feature set was optimized and screened by the RFE method. In order to effectively predict the risk of smoking-induced lung cancer through these features, the SVM method [20] was used to train the model. The linear fitting method was adopted. Five-fold cross-validation method was used [21] by which samples were divided into five parts after random sorting. Four of them were used as training sets to construct the SVM model, and the remaining one was predicted to calculate accuracy. Above process was repeated 5 times until each one was predicted as a prediction set for once only. Results of the 5 predictions were plotted as ROC curves, and the AUC values were calculated to evaluate the predicted performance of the SVM model.

## Results

### DEGs extraction

**Fig 1:**
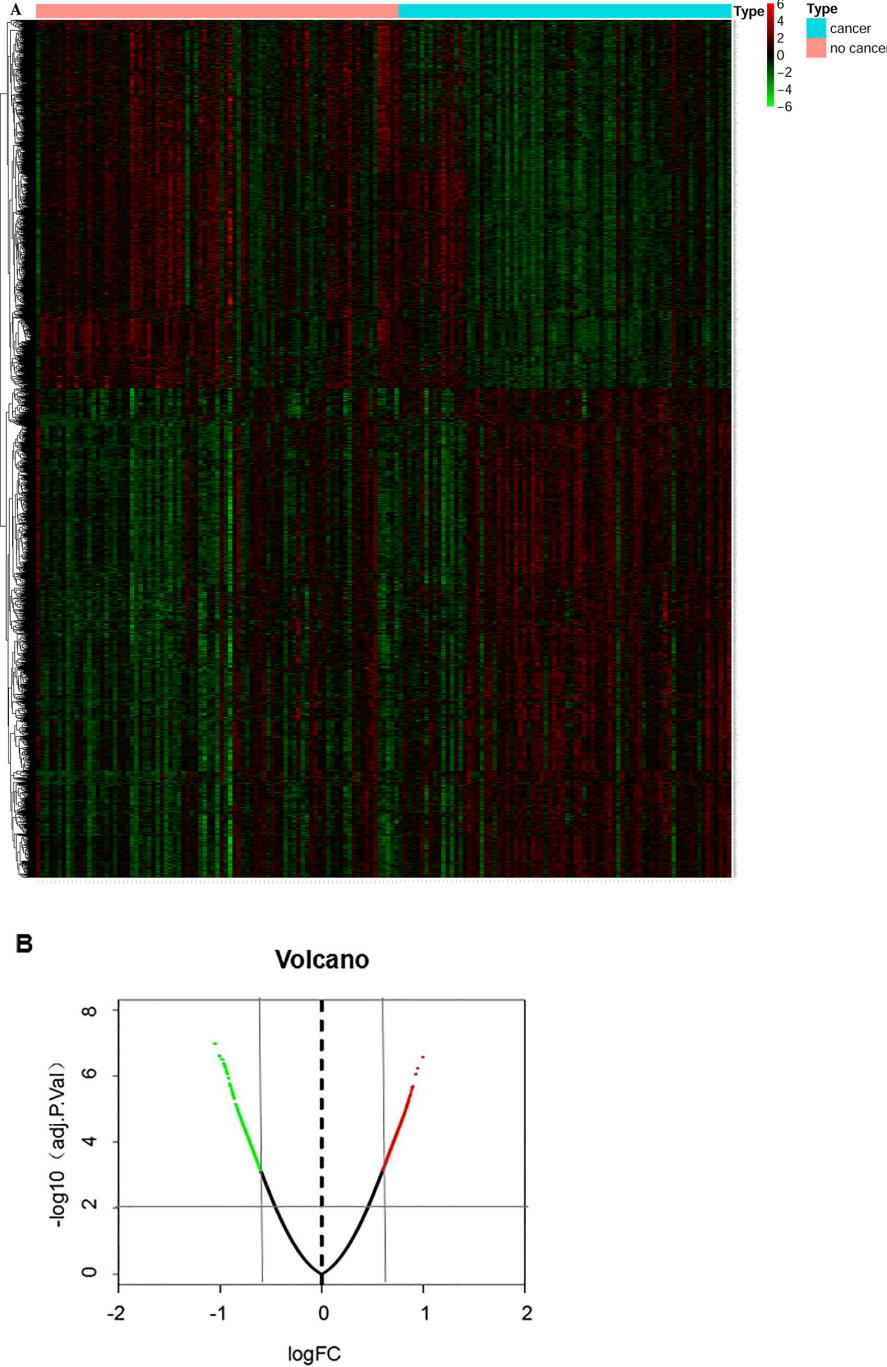
Distribution of DEGs expression in lung cancer smokers and non-lung cancer smokers. A: Heat map of DEGs; B: Volcano plot of DEGs. Red represents up-regulated genes, green represent down-regulated genes in these two maps.

85 non-lung cancer smokers were served as control group and 78 lung cancer smokers were served as case group to identify DEGs. Genes with adjusted *P*<0.01 and |logFC|>0.6 were selected as the DEGs. Ultimately, 1923 DEGs were obtained, of which 826 were down-regulated and 1098 were up-regulated (Fig 1) (For detailed data, see supplementary material DEG.txt).

### Unsupervised hierarchical clustering analysis

**Fig 2:**
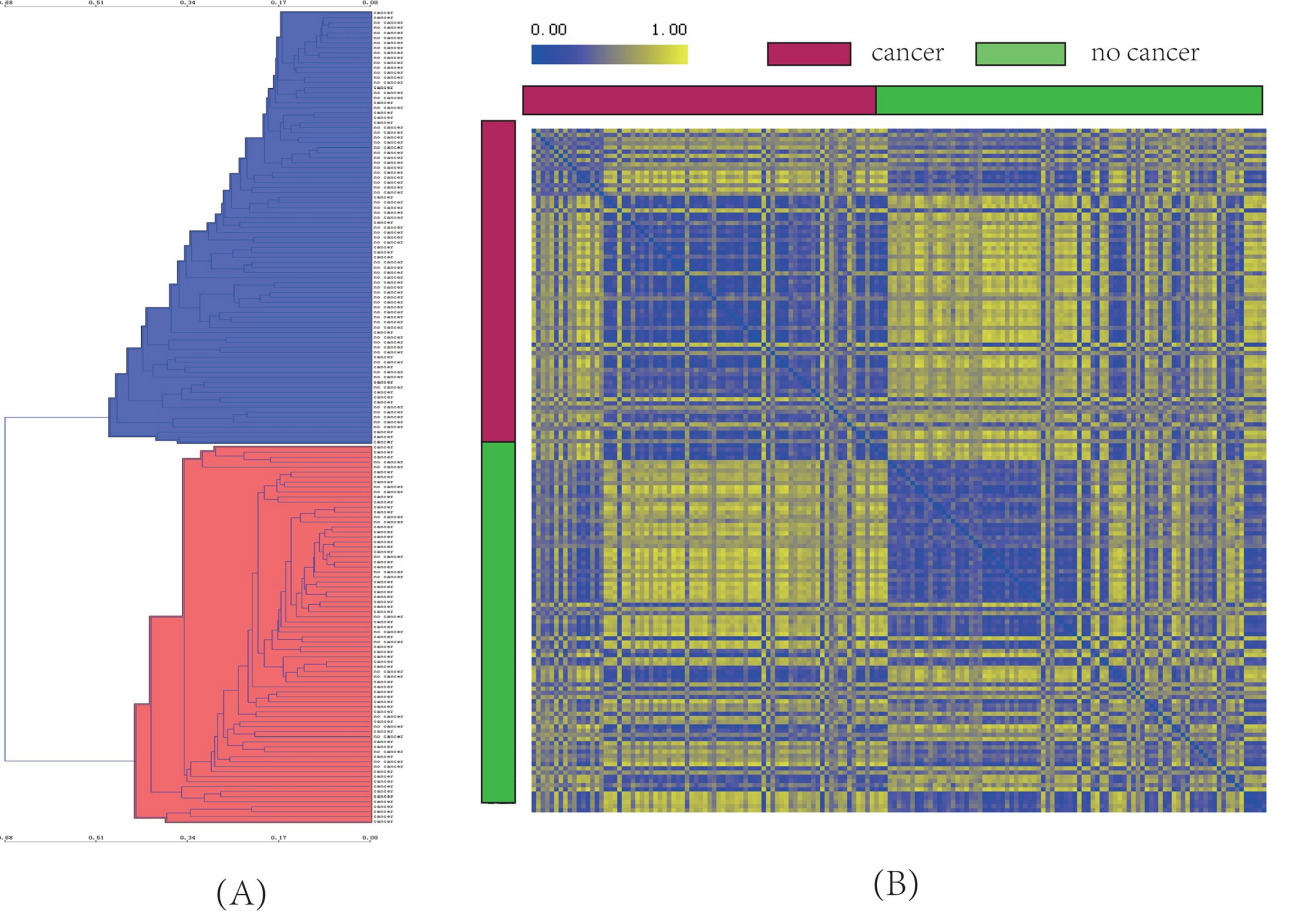
Clustering results of 163 smokers. (A) Unsupervised hierarchical clustering results, blue represents predomination of non-lung cancer, red represents domination of lung cancer; (B) Distance map of lung cancer samples and non-lung cancer samples. The closer the distance, the closer the color is to blue; the further the distance, the closer the color is to yellow.

In order to validate the efficacy of DEGs in distinguishing the two kinds of smokers and identify the similarity of the two smoker populations, unsupervised hierarchical clustering method was used to analyze the 163 samples. The result was shown in Fig 2A. The two categories of clustering results were marked as red and blue, respectively. The non-lung cancer smokers accounted for 75% of the total blue clusters and lung cancer smokers accounted for 74% of the total red cluster samples. We used the confusion matrix to statistically predict the accuracy of each component in hierarchical clustering (as listed in Table 1). The rows in table represented observation value, and columns represented cluster prediction value. There were 78 lung cancer samples and 85 non-lung cancer samples in observation samples. While in the cluster samples, 76 samples were classified into the cancer group, of which 56 were correctly classified with an accuracy of 74%. In addition, 87 samples were classified as non-cancer group, of which 65 were correctly classified with an accuracy of 75%.

**Table 1:** Confusion matrix.

		clustered sample	
	group	Cancer	no cancer
observed sample	cancer	56	22
	no cancer	20	65

To further evaluate the correlation and distance between the two sets of samples, we visualized the distance between two random samples in Fig 2B. It could be seen that the distance within the group were closer together while the distance between the groups was far. Therefore, there is a genetic difference between lung cancer smokers and non-lung cancer smokers, which means that these genes have different functions.

### Functional enrichment analysis

In order to further analyze the biological functions regulated by DEGs between lung cancer smokers and non-lung cancer smokers at functional level, GO and KEGG enrichment analyses were performed on DEGs. In order to analyze the directionality of abnormal changes in functional levels, we enriched the up-regulated DEGs and down-regulated DEGs, respectively, with the significance P value as 0.05. The enrichment results were shown in Tables 2–5. Up-regulated DEGs were mainly involved in the regulation of protein, mRNA anabolism and energy metabolism-related functions. Down-regulated DEGs were mainly involved in the regulation of cell signaling processes, including membrane potential regulation, second messenger, intracellular signaling, apoptosis, immunity and other related functions. Smoking induces the disorder of protein, mRNA anabolism and energy metabolism in human body, resulting in the abnormal function of normal cells. At the same time, abnormal function of apoptotic process and abnormal immune system mediated by immune cells such as T cells and NK cells, are also important causes of carcinogenesis.

**Table 2:** GO enrichment using up-regulated genes.

Term	Count	*P Value*
GO:0044267~cellular protein metabolic process	250	9.04E-16
GO:0015031~protein transport	100	5.37E-11
GO:0006886~intracellular protein transport	60	4.02E-10
GO:0016070~RNA metabolic process	104	2.08E-07
GO:0043632~modification-dependent macromolecule catabolic process	70	1.09E-06
GO:0030163~protein catabolic process	73	2.57E-06
GO:0051246~regulation of protein metabolic process	66	3.14E-06
GO:0006396~RNA processing	66	3.47E-06
GO:0044257~cellular protein catabolic process	70	6.25E-06
GO:0016071~mRNA metabolic process	48	1.43E-05
GO:0006397~mRNA processing	41	9.44E-05
GO:0006412~translation	41	1.83E-04
GO:0006464~protein modification process	131	2.20E-04
GO:0006259~DNA metabolic process	54	6.85E-04
GO:0032268~regulation of cellular protein metabolic process	50	0.001416
GO:0042981~regulation of apoptosis	75	0.002679
GO:0043067~regulation of programmed cell death	75	0.003472
GO:0010605~negative regulation of macromolecule metabolic process	68	0.005107
GO:0031325~positive regulation of cellular metabolic process	79	0.00553

**Table 3:** KEGG enrichment using up-regulated genes.

Term	Count	*P Value*
hsa00020: Citrate cycle (TCA cycle)	10	2.33E-04
hsa04120: Ubiquitin mediated proteolysis	23	2.74E-04
hsa04142: Lysosome	19	0.001623
hsa03040: Spliceosome	19	0.003749
hsa00510: N-Glycan biosynthesis	10	0.004849
hsa03018: RNA degradation	11	0.006855
hsa00190: Oxidative phosphorylation	18	0.011546
hsa05210: Colorectal cancer	13	0.016526
hsa05016: Huntington's disease	22	0.018547
hsa05012: Parkinson's disease	16	0.041203
hsa00230: Purine metabolism	18	0.048018

**Table 4:** GO enrichment using down-regulated genes.

Term	Count	*P Value*
GO:0009887~organ morphogenesis	44	6.00E-04
GO:0019932~second-messenger-mediated signaling	23	0.001072
GO:0030808~regulation of nucleotide biosynthetic process	14	0.001434
GO:0007242~intracellular signaling cascade	80	0.001696
GO:0006140~regulation of nucleotide metabolic process	14	0.002324
GO:0045761~regulation of adenylate cyclase activity	12	0.004139
GO:0048705~skeletal system morphogenesis	13	0.00487
GO:0042391~regulation of membrane potential	14	0.007974

**Table 5:** KEGG enrichment using down-regulated genes.

Term	Count	*P Value*
hsa04080: Neuroactive ligand-receptor interaction	26	5.02E-04
hsa04650: Natural killer cell mediated cytotoxicity	16	0.001677
hsa04512: ECM-receptor interaction	11	0.006667
hsa04660: T cell receptor signaling pathway	12	0.014253
hsa04912: GnRH signaling pathway	11	0.018809
hsa04020: Calcium signaling pathway	16	0.021719
hsa05340: Primary immunodeficiency	6	0.024776
hsa04210: Apoptosis	9	0.047056
hsa04510: Focal adhesion	16	0.049391

### PPI network construction

**Fig 3:**
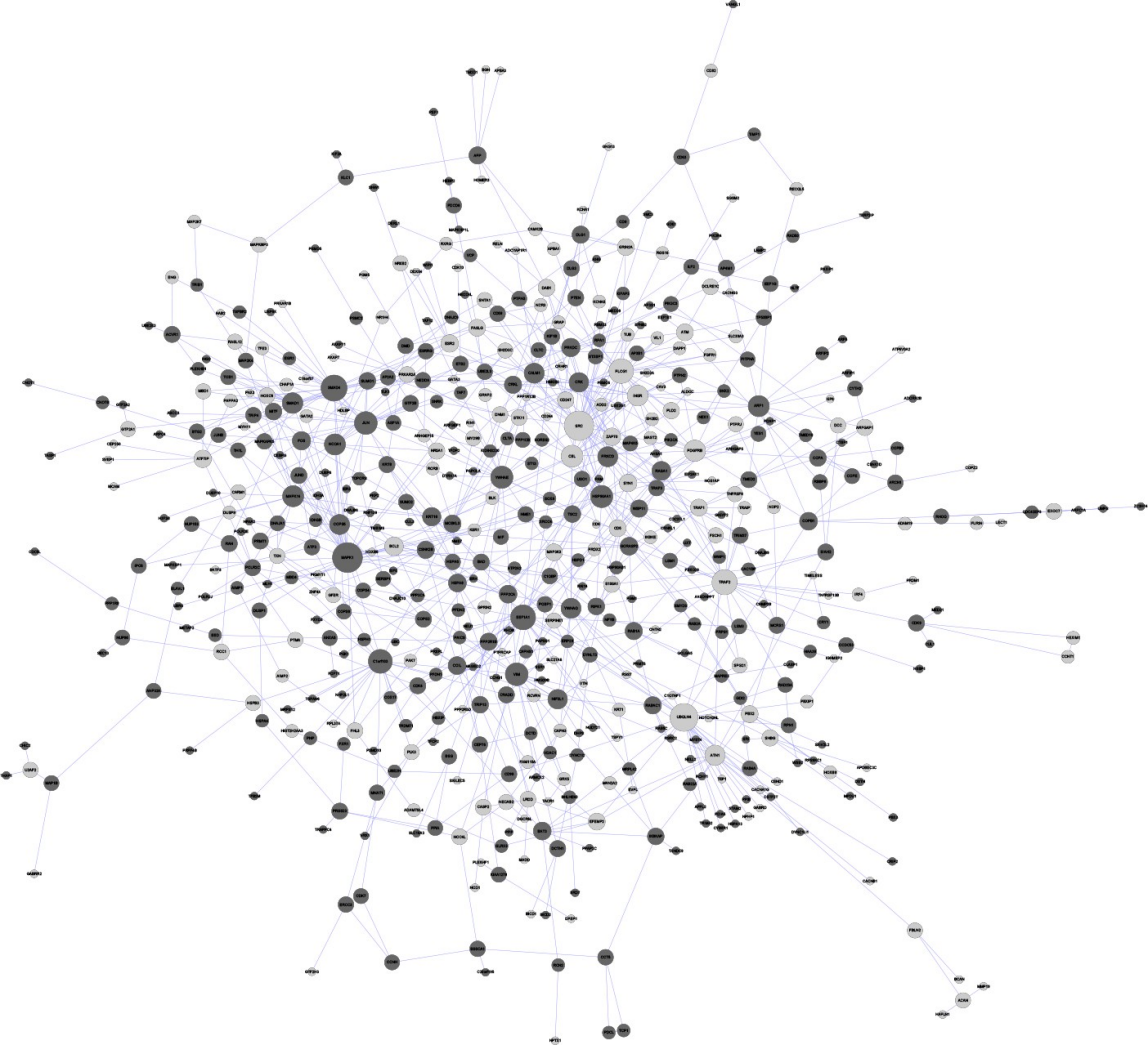
PPI network.

Disease-related genes are not relatively independent, since they often participate in the regulation of important biological processes by interacting with each other. Meanwhile, if a gene has more adjacent gene nodes in the process of system regulation and interaction, it tends to be more important. Therefore, we mapped the DEGs to the human PPI, constructed the network through cytoscape software and performed topological analysis on the network to find important genes with high node degree. The degree distribution of nodes in the network was used as a weight to evaluate their contribution rate and effect, and systematically analyze the role of DEGs of lung cancer smokers and non-lung cancer smokers in biological networks. Results were shown in Fig 3, the network contained a total of 774 nodes, of which 496 genes were up-regulated (indicated by dark black nodes) and 278 genes were down-regulated (indicated by light-colored nodes). Next, we used the network analysis plug-in to analyze the topological properties of the network. The node size is used to represent the size of the node degree distribution. The larger the node is, the greater the node degree directly interacted with the node is in the network, and the more adjacent genes are affected by it. Detailed node degree distribution data can be found in the supplementary material Degree.txt.

### Functional deviation score matrix

**Fig 4:**
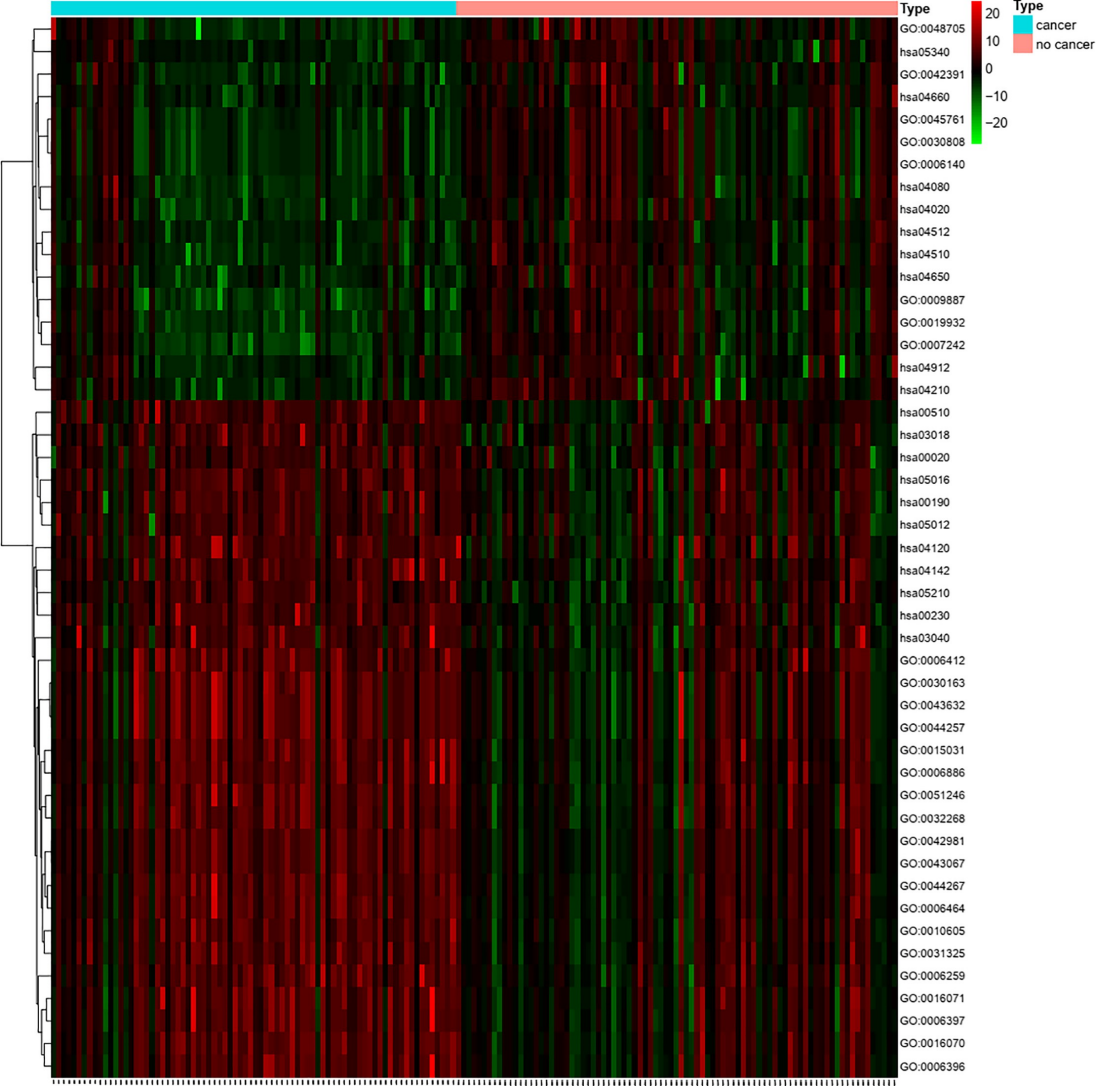
Functional score heat map for each sample. (The closer to red, the higher the score; the closer to green, the lower the score.).

The biological functions of significant functional terms (including GO term and KEGG pathway) obtained by enrichment analysis were regulated by multiple DEGs. Abnormally expressed DEGs involved in the regulation of these functions would be accompanied with abnormal functional levels of these functional terms. According to the calculation formula of functional deviation score, we used the enriched DEGs expression value in 47 functions (GO or KEGG term) and the deviation from the expression mean of the control group to calculate the Euclidean distance, thus obtaining the functional score corresponding to each sample and finally constructing a matrix (The specific scoring was shown in Fig 4 and the supplementary material path score.txt).

### Recursive features elimination feature selection

**Fig 5:**
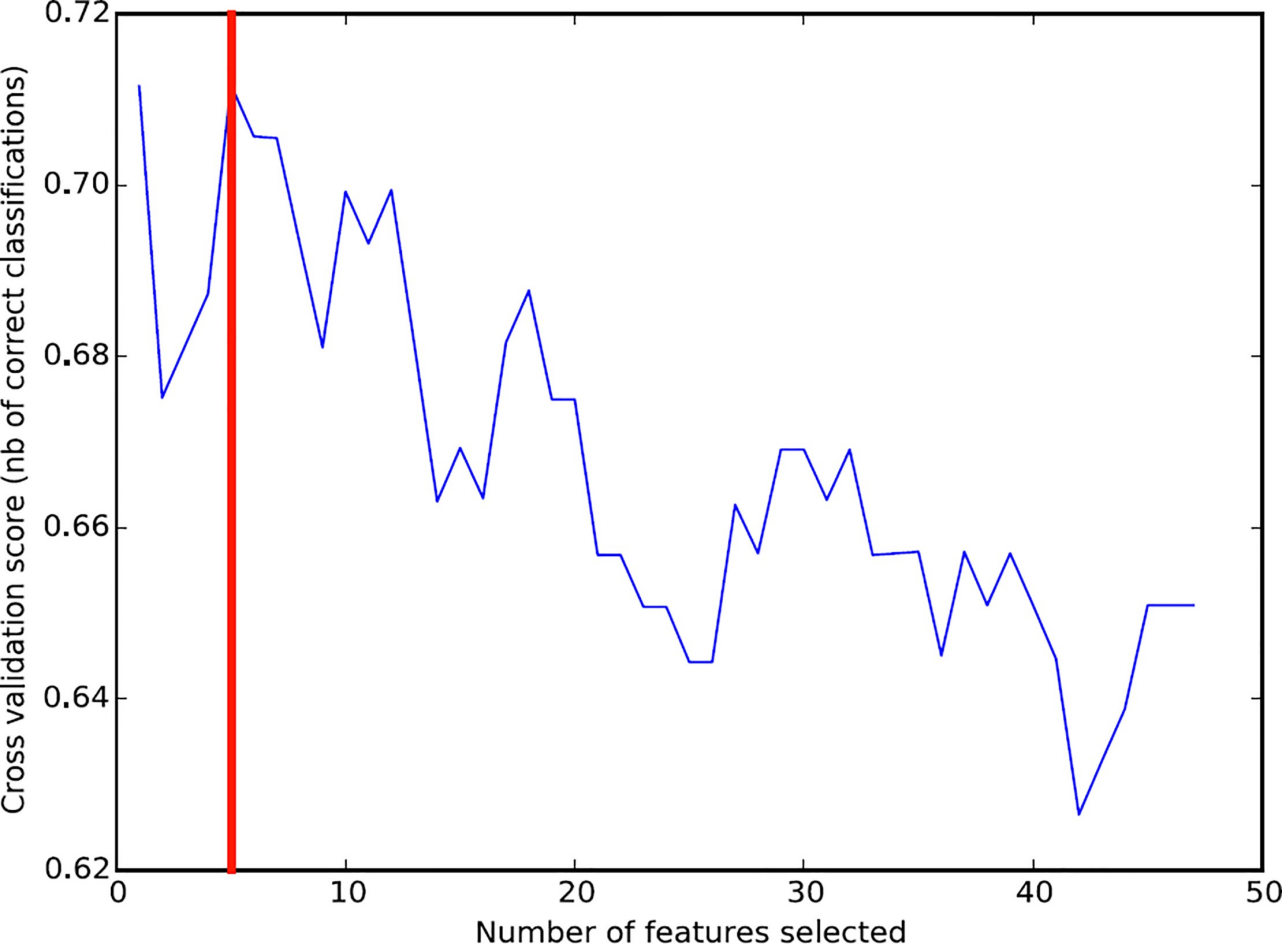
Feature recursive optimization. The horizontal axis is the number of feature selections, and the vertical axis is the prediction accuracy of cross-validation.

RFE method was used to optimize and screen functional features from functions with various changes that could be diagnosed and predicted effectively in advance before the occurrence of lung cancer. The results were shown in Fig 5, the accuracy represented by blue lines changed as the number of feature sets changed. It could be observed that when the number of feature sets was 5 (corresponding to the red line in the figure), the RFE prediction accuracy was the highest. That was to say, the top 5 most significant function features could make the classifier perform best. These five features were GO: 0007242~intracellular signaling cascade, GO:0006140~regulation of nucleotide metabolic process, hsa04080: Neuroactive ligand-receptor interaction, hsa04120: Ubiquitin mediated proteolysis, hsa04142: Lysosome.

### SVM classifier construction

**Fig 6:**
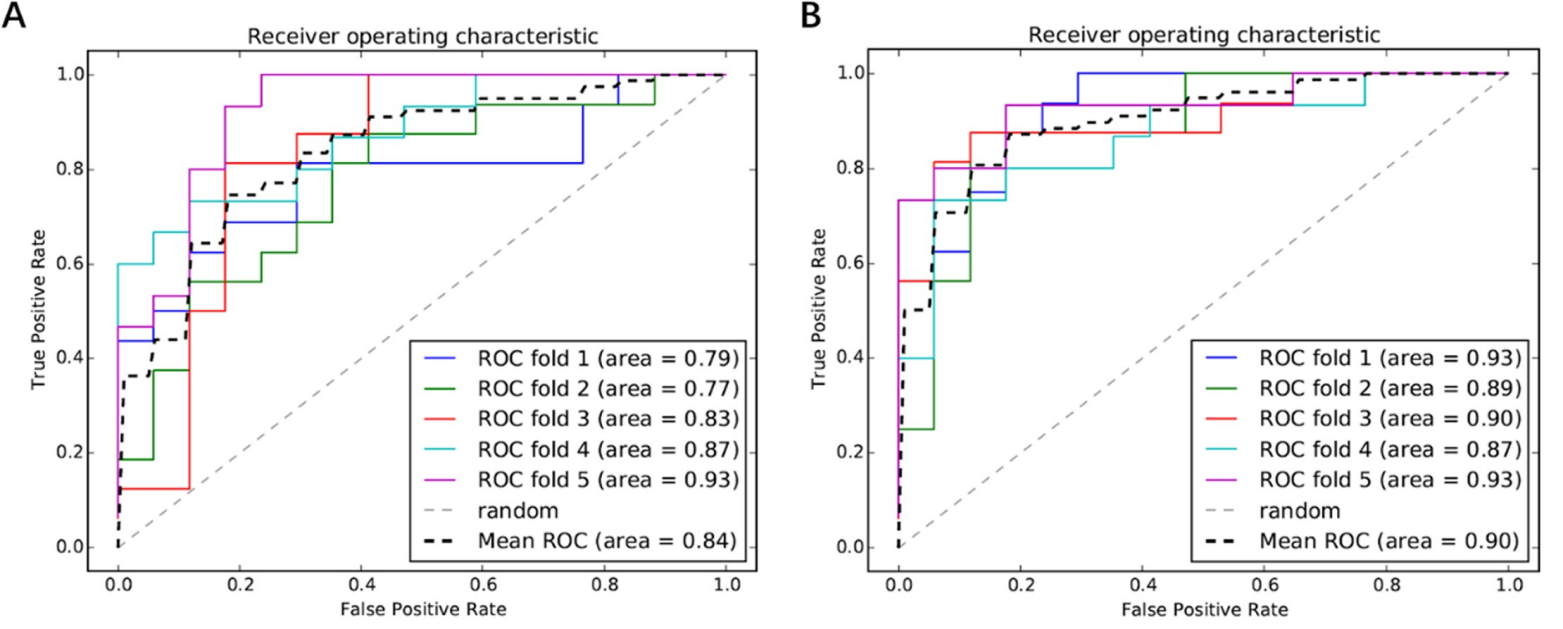
ROC curves for evaluating the performance of the SVM classifier. A: ROC curves without clinical features, 1–4 is training set and 5 is test set; B: ROC curves with clinical features, 1–4 is training set and 5 is test set.

We used five functional features to randomly rearrange all lung cancer smokers and non-lung cancer smokers. Five-fold cross-validation was conducted to plot ROC curves, and the classification accuracy was evaluated through AUC value. The results were shown in Fig 6A, and the average AUC value was 0.84.

In order to analyze the efficacy of clinical diagnostic indicators (age, gender, smoke age, smoking index, lymphatic lesions, nodule size) for the diagnosis of smoking-induced lung cancer, these indicators were used as new features and combined with 5 functional features to perform classified prediction again. Afterwards, a total of 11 features were obtained, which were used as classification criteria. The ROC results were shown in Fig 6B. After the combination of clinical features, the accuracy increased from 84% to 90%, indicating that clinical indicators had a certain auxiliary effect on the risk of smoking-induced lung cancer. Smokers with longer smoking history, larger nodules and lymphadenopathy are more likely to develop lung cancer than other smokers.

## Discussion

Smoking is a major risk factor for lung cancer [22]. Inhalation of second-hand smoke or environmental tobacco smoke increases the risk of lung cancer even if people do not smoke [2]. However, not all smokers are diagnosed with lung cancer, so it is of great significance to find the feature risk pathways of smoking carcinogenesis for the early diagnosis of lung cancer in smokers and realize personalized treatment for smoking patients. He X *et al*. have performed meta-analysis of mRNA in lung adenocarcinoma (LUAD) smokers and non-smokers and have explored five genes related to LUAD [23]. But there are still few studies on DEGs in smokers and non-smoking patients. In present study, we compared and screened DEGs of the lung cancer smokers and the non-lung cancer smokers. These genes play an important role in the development of lung cancer, and further exploration of the biological functions and their changing directions regulated by these genes could help us to discover new diagnostic markers.

In order to analyze the directionality of functional changes, we took the up-regulated and down-regulated genes for enrichment analysis. The results indicated that there was a significant difference between the up-regulated biological functions and the down-regulated biological functions. Up-regulated functions focused on regulating protein and mRNA anabolism, as well as energy metabolism-related function. Down-regulated DEGs were mainly involved in the regulation of cell signaling processes, including membrane potential regulation, second messenger, intracellular signaling and apoptosis, immunity and other related functions.

We used DEGs to perform unsupervised hierarchical clustering analysis on the two groups of samples, and obtained two main clustering results, which were predominated by lung cancer smokers and non-lung cancer smokers with accuracy of 74% and 75%, respectively. The results suggested that at the molecular level, two groups with different risk of lung cancer could be distinguished to a certain extent, but the accuracy did not meet the clinical diagnostic requirements. This might due to the fact that the genes are more sensitive to environmental stimulus, so we considered converting genes into functional analysis for more robust prediction.

In recent years, more and more researches have been conducted to verify the accuracy of tumor biomarker prediction based on SVM classifiers [12, 24, 25]. In this study, we identified five important biological functions through RFE which included GO: 0007242~intracellular signaling cascade, GO:0006140~regulation of nucleotide metabolic process, hsa04080: Neuroactive ligand-receptor interaction, hsa04120: Ubiquitin mediated proteolysis, hsa04142: Lysosome. These signaling pathways are closely related to cell metabolism and signal transduction. The accuracy of using these five functions for pre-SVM classifier prediction is up to 84%, which is much higher than the previous result of unsupervised hierarchical clustering analysis. It is proved that the analysis at functional level analysis is more stable than that at the genetic level. These significantly altered biological functions identify different risk degrees associated with different types of smoking-induced lung cancers. The mechanisms that affect the cancer risk are caused by these functional changes.

We further combined five significant functional features with clinical diagnostic indicators, including age, gender, smoking index, smoking age, lymphatic lesions and tumor size, etc. The accuracy of classification prediction was improved to 90% by encoding and merging the clinical diagnostic indicators into a new feature set, indicating that the clinical diagnostic indicators also had certain discriminative performance. Therefore, combining clinical indications and changes in biological functions is of great significance for early diagnosis and prediction of smoking-induced lung cancer risk. The human-behavior indicators are country specific and SVM has an excellent power in predicting their lung cancer mortality (CM) with the average accuracy in prediction of 96.08% for the 13 countries tested between 2014 and 2016 [26]. Therefore, SVM has a good application prospect to identify the characteristic risk pathways of smoking induced lung cancer.

From this study, it can be concluded that smoking may be an important cause of lung cancer but not the only factor. There are still more complex stress and self-protection mechanisms in living organisms. Under the stimulation of nicotine, the body initiates a series of biological functions to counteract the cancerization process. This complex process is one of the reasons why some smokers do not have cancer. However, when this stress response is absent or decompensated, the cancerization predominates and smokers have a significantly increased risk of developing lung cancer. Based on the expression of gene level, we have classified clinically smoked with different cancer risk samples, and identified five feature risk pathways that can be used to identify smoking-induced lung cancer. It provides basis for early diagnosis and accurate treatment of lung cancer smoking patients. However, its specific biological mechanism still needs further exploration.

## Supporting information

S1 File (ZIP)
