# Correction: Identification of feature risk pathways of smoking-induced lung cancer based on SVM

**DOI:** 10.1371/journal.pone.0235854

**Published:** 2020-07-01

**Authors:** Rongjun Chen, Jinhui Lin

There is an error in affiliation 1 for author Rongjun Chen. The correct affiliation 1 is: Department of Surgery, Anji County Third People’s Hospital, Zhejiang China.
